# Effects of Combined vs. Single-Source Supportive Communication from Parents and Coaches on Mental Health and Self-Regulation in Adolescent Football Players

**DOI:** 10.3390/ejihpe16030033

**Published:** 2026-02-27

**Authors:** Ameni Essid, Mohamed Mansour Bouzourraa, Hajer Sahli, Wissem Dhahbi, Achraf Ammar, Khaled Trabelsi, Mohamed Jarraya, Makram Zghibi

**Affiliations:** 1 High Institute of Sport and Physical Education of Sfax, University of Sfax, Sfax 3000, Tunisia; ameni.essid@isseps.usf.tn (A.E.); trabelsikhaled@gmail.com (K.T.); mohamed.jarraya@isseps.usf.tn (M.J.); 2 Research Laboratory Education, Motricité, Sport et Santé, EM2S, LR19JS01, High Institute of Sport and Physical Education of Sfax, University of Sfax, Sfax 3000, Tunisia; 3 High Institute of Sports and Physical Education of Kef, University of Jendouba, Kef 7100, Tunisia; bouzouraamansour@gmail.com (M.M.B.); hajer.sahli@issepkef.u-jendouba.tn (H.S.); wissem.dhahbi@issepkef.u-jendouba.tn (W.D.); makrem.zghibi@issepkef.u-jendouba.tn (M.Z.); 4 Research Unit “Sport Sciences, Health and Movement”, High Institute of Sports and Physical Education of Kef, University of Jendouba, Kef 7100, Tunisia; 5 Training Department, Qatar Police Academy, Police College, Doha 7157, Qatar; 6 Department of Training and Movement Science, Institute of Sport Science, Johannes Gutenberg-University Mainz, 55122 Mainz, Germany; 7 Research Laboratory, Molecular Bases of Human Pathology, LR19ES13, Faculty of Medicine of Sfax, University of Sfax, Sfax 3000, Tunisia; 8 Department of Movement Sciences and Sports Training, School of Sport Science, The University of Jordan, Amman 28002, Jordan

**Keywords:** supportive communication, emotional health, self-regulation, adolescent players

## Abstract

Background: Adolescent football players are exposed to substantial psychological demands, and psychosocial support within family and sport environments has been shown to influence motivational climate, stress responses, and emotional well-being. However, the comparative effects of coordinated multi-source psychosocial support from parents and coaches versus single-source support on psychological outcomes remain insufficiently examined. Aims: to examine the effects of a multi-component psychosocial program involving parents and coaches on depression, anxiety, stress, and self-regulation in adolescent football players. Methods: A total of 60 male adolescent soccer players were recruited and randomly assigned to four groups: combined supportive communication from both parents and coaches (SCCP; n = 15), supportive communication from coaches only (SCC; n = 15), supportive communication from parents only (SCP; n = 15), and a control group (CG; n = 15). Over a 12-week intervention period, participants attended 12 sessions incorporating structured supportive communication and related psychosocial strategies. Mental health outcomes were assessed using the Depression Anxiety Stress Scale (DASS-21), while self-regulation was measured with the Self-Regulation Questionnaire (SRQ). Results: The SCCP group demonstrated the largest improvements across outcomes, with reductions observed in anxiety, depression, and stress, and significant time effects for all mental health parameters. For depression, the time effect showed a (*p* < 0.001; η^2^p = 0.93 [Very large]; −45.77%). Anxiety scores also indicated a significant time effect (*p* < 0.001; η^2^p = 0.81 [Very large]; −46.55%). Stress demonstrated an exceptionally significant time effect (*p* < 0.001; η^2^p = 0.98 [Very large]; −48.29%). Additionally, the self-regulation outcomes showed significant improvements, with a time effect for the Planning subscale of the SRQ indicating (*p* < 0.001; η^2^p = 0.86 [Very large]; +66.38%). Conclusion: The findings provide preliminary evidence that a multi-component program involving parents and coaches is associated with improvements in depression, anxiety, stress, and self-regulation among adolescent football players. These results suggest that coordinated psychosocial support across family and sport environments may contribute to adolescent athletes’ psychological well-being, although replication in other cultural and sporting contexts is required.

## 1. Introduction

Adolescence is a critical developmental period characterized by rapid physical, emotional, and cognitive changes (World Health Organization, 2024b). During this time, individuals are particularly vulnerable to mental health challenges, including depression, anxiety, and stress (Kessler et al., 2015). Research indicates that around 10–20% of adolescents experience mental health disorders globally, with depression and anxiety among the most prevalent conditions (Keeley, 2021). The World Health Organization reported that globally, one in seven adolescents 14% experience mental disorders, with anxiety and depression leading as common conditions (World Health Organization, 2024a). Additionally, the COVID-19 pandemic has exacerbated these issues, with post-pandemic reports indicating higher rates of clinically significant depression (23.8%) and anxiety (19%) (Benton et al., 2021; World Health Organization, 2022). These mental health challenges can have long-lasting effects on social functioning, academic performance, and overall well-being, particularly if not addressed early.

In recent years, there has been growing interest in understanding how organized sports, such as football (soccer), can support adolescent development, particularly in relation to mental health (Zhao et al., 2025). Football, one of the most popular sports worldwide, is not only a platform for physical activity but also a significant avenue for social and psychological development (Canter et al., 2024). Participation in team sports has been associated with numerous benefits, including improved self-esteem, social skills, and emotional regulation (Eime et al., 2013; Merkel, 2013). However, it is also important to acknowledge that the competitive nature of sports can introduce significant stressors, such as performance anxiety, fear of failure, and pressure from both parents and coaches (Coutinho et al., 2024). These stressors can negatively impact mental health if not adequately managed (Vella et al., 2017).

Positive supportive communication, defined as the specific verbal and behavioral ways in which parents and coaches convey encouragement, guidance, and emotional validation, is one of the most crucial factors shaping adolescents’ emotional and psychological experiences in sport (Coleman & Eys, 2024; Zhang et al., 2025). While social support reflects the broader relational environment surrounding young athletes, supportive communication captures the day-to-day interactional processes through which adolescents learn to cope with challenges, regulate emotions, and interpret performance feedback.

Parents, as primary caregivers, play a central role in providing emotional support and guidance during adolescence (Son & Kim, 2024), and the quality of parent–adolescent communication strongly influences how young athletes cope with sport-related demands. Research shows that supportive and encouraging parental communication can buffer the negative effects of stress, promote adaptive coping, and foster a sense of competence, whereas critical or overly demanding communication can increase anxiety, undermine self-esteem, and contribute to feelings of inadequacy (Zapf et al., 2024).

Similarly, the coach–athlete relationship plays a pivotal role in shaping an adolescent athlete’s mental health and performance (Charlina et al., 2024). Coaches serve as authority figures and mentors who can significantly influence an athlete’s emotional well-being and self-regulation skills. Studies have consistently shown that positive feedback, clear communication, and emotional support from coaches can enhance athletes’ self-confidence, reduce stress, and improve their ability to regulate emotions under pressure (Bergmann et al., 2024; Donnelly et al., 2024). Conversely, negative or authoritarian communication styles can contribute to increased anxiety, stress, and burnout among young athletes (Liu & Li, 2024).

Adolescent football players are particularly affected by these dynamics, given the unique demands of the sport (McBurnie et al., 2021). Soccer is a fast-paced, high-pressure environment that requires players to make quick decisions, maintain composure, and regulate emotions effectively. Players must balance individual performance with team dynamics, manage physical and emotional exhaustion, and cope with the expectations of coaches, teammates, and parents. These pressures can contribute to heightened anxiety and stress, particularly if adolescents lack adequate coping mechanisms and support systems (Domínguez-González et al., 2024). In this context, Self-regulation, or the ability to manage one’s emotions, behaviors, and thoughts in the face of external demands, is a critical skill for soccer players (Ruiz et al., 2021). Effective self-regulation allows players to maintain focus during a match, manage frustration after mistakes, and stay composed under pressure. Studies have shown that athletes with strong self-regulation skills are more likely to succeed in high-pressure situations, recover from setbacks, and maintain their mental well-being (Hamoud et al., 2024; Mcgreary et al., 2024). Positive communication from both parents and coaches plays a key role in fostering these skills, providing athletes with the emotional tools needed to cope with the demands of the sport (Burke et al., 2024; Sulistiyono et al., 2024).

From a theoretical perspective, the effects of combined parental and coach communication can be understood through an integration of social support theory (Cohen & Wills, 1985) and models of emotion and self-regulation (Morris et al., 2007; Cui et al., 2014). Parents significantly shape the coach–athlete relationship by providing information, opportunities, and emotional support, thereby influencing triadic communication and fostering adolescents’ self-regulation and coping (Jowett & Timson-Katchis, 2005).

Supportive environments are thought to buffer stress by shaping how adolescents appraise challenges, regulate emotions, and develop confidence in their coping abilities. When both parents and coaches provide consistent guidance and emotional validation, adolescents may experience more stable regulatory cues across home and sport contexts, strengthening self-regulatory capacity and psychological resilience.

While the individual effects of parental and coach communication on adolescent athletes have been well-documented, there is limited research exploring the combined influence of these two key figures. Adolescents spend significant time both at home and in sports environments, and the consistency of messages received from parents and coaches may be critical in shaping their mental health outcomes. However, inconsistent or conflicting communication between parents and coaches may have the opposite effect, creating confusion and increasing stress for young athletes. For instance, a coach who emphasizes performance may undermine a parent’s efforts to promote emotional well-being and balance, leading to increased pressure and anxiety in the athlete. Understanding the dynamics of this interaction is crucial for developing strategies to optimize the mental health and performance of adolescent athletes. Although previous research has examined parent–coach–athlete triads, most studies have focused on motivational climate, performance, or sport socialization rather than on how coordinated supportive communication across these two social agents influences mental health and self-regulation. The present study addresses this gap by examining whether consistency of supportive communication across home and sport contexts is associated with adolescent psychological outcomes.

Given the importance of both parental and coach communications in shaping adolescent mental health and self-regulation, this study aims to explore the combined effects of supportive communication delivered by parents and coaches on depression, anxiety, stress, and self-regulation in adolescent soccer players. In this context, we hypothesized that adolescents receiving combined supportive communication from both parents and coaches would exhibit (a) lower levels of depression, anxiety, and stress, and (b) higher levels of self-regulation than those receiving single-source support or no intervention. This research seeks to fill the gap in the existing literature by investigating the interaction between parental and coach communication and its impact on the mental health outcomes of adolescent soccer players.

## 2. Materials and Methods

### 2.1. Participants

First, seventy-six (n = 76) adolescent male soccer players were initially recruited from 4 different football clubs competing in the Tunisian Championship in the North-West region of Tunisia during the 2023/2024 competitive season. We used this strategy to enhance the external validity and generalizability of the findings by reducing the influence of club-specific training cultures, coaching styles, and organizational environments. All clubs competing at a comparable competitive level (amateur Ligue 3) and followed similar seasonal calendars, ensuring homogeneity in competitive demands and allowing meaningful between-group comparisons and to minimize potential clustering effects and selection bias (Campbell & Stanley, 2015; Hox et al., 2017; Thomas et al., 2023).

Inclusion criteria were: (i) male soccer players aged 15–17 years, (ii) minimum of three years of organized competitive soccer experience, (iii) regular participation in training (≥3 sessions per week), and (iv) medical clearance for high-intensity physical activity. Exclusion criteria included: (i) musculoskeletal injury or illness within the previous six months, (ii) diagnosed psychological, neurological, or developmental disorders, (iii) use of medication affecting physical or psychological performance, and (iv) absence from more than two training sessions during the intervention period.

**Figure 1:**
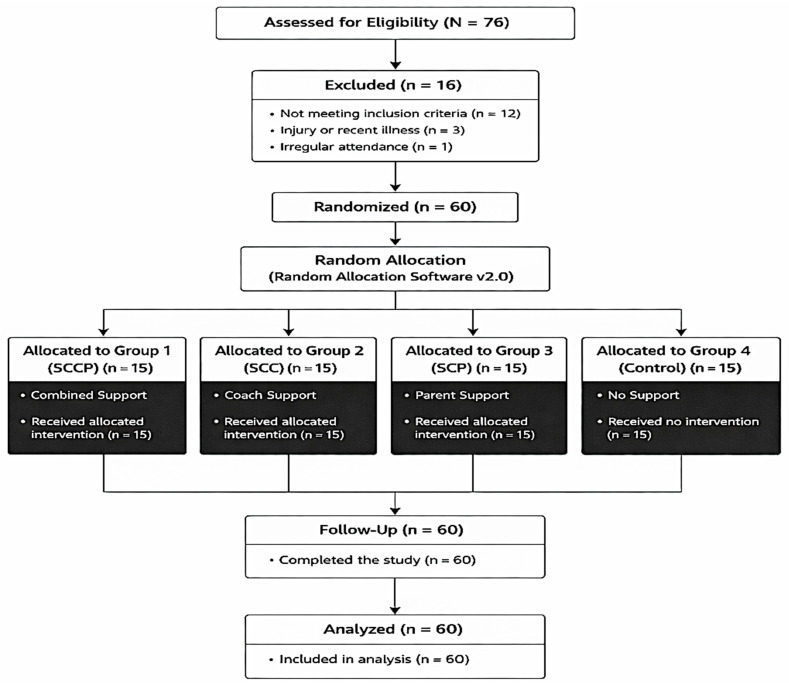
CONSORT flow diagram illustrating participant recruitment, eligibility screening, exclusions, computer-generated randomization, group allocation, follow-up, and final analysis of adolescent male soccer players, reported in accordance with CONSORT 2010 guidelines for randomized controlled trials (Schulz et al., 2010).

Following eligibility screening based on predefined inclusion and exclusion criteria, sixty players met the study requirements and completed the intervention, while sixteen players were excluded (injury history, irregular attendance and failure to meet eligibility criteria). The eligible participants were randomly allocated into four groups using the Random Allocation Software 2.0 to ensure allocation concealment and reduce selection bias (Figure 1).

Group 1 (SCCP; n = 15; Age = 16.2 ± 1.1 years; Height = 172.5 ± 5.4 cm; Weight = 65.1 ± 4.8 kg; BMI = 21.8 ± 1.2 kg/m^2^) received combined supportive communication from both coaches and parents. Group 2 (SCC; n = 15; Age = 16.0 ± 1.2 years; Height = 170.3 ± 6.1 cm; Weight = 63.8 ± 5.0 kg; BMI = 21.7 ± 1.3 kg/m^2^) received supportive communication exclusively from coaches. Group 3 (SCP; n = 15; Age = 16.1 ± 1.0 years; Height = 171.7 ± 5.9 cm; Weight = 64.5 ± 4.5 kg; BMI = 21.8 ± 1.0 kg/m^2^) received supportive communication exclusively from parents, while Group 4 (CG; n = 15; Age = 16.3 ± 1.3 years; Height = 172.1 ± 5.6 cm; Weight = 65.0 ± 4.6 kg; BMI = 21.9 ± 1.1 kg/m^2^) served as the control group and received no specific communication-based intervention.

An a priori power analysis (G*Power 3.1.9.3, Heinrich Heine Universität Düsseldorf, Germany) indicated that a minimum of 15 participants per group was required to detect a large effect size (d = 0.97), with a statistical power of 0.90 and an alpha level of 0.05 (Faul et al., 2009). Such a priori calculations are recommended to ensure adequate sensitivity in detecting meaningful effects in experimental and quasi-experimental studies (Button et al., 2013; Lakens, 2021). The study was conducted in accordance with the ethical principles outlined in the Declaration of Helsinki (World Medical Association, 2013), and all procedures received approval from the institutional ethics committee to protect participant welfare (Resnik, 2018).

Written informed consent was obtained from all participants and their legal guardians. Confidentiality, voluntary participation, and the right to withdraw at any time were ensured. Ethical approval was obtained from the institutional review board of the Higher Institute of Sport and Physical Education of Kef (ISSEPK-0016/2025).

The study was conducted exclusively with male players to limit biological and maturational variability associated with sex-related differences during adolescence, particularly regarding hormonal fluctuations, growth velocity, emotional regulation, and psychophysiological responses to psychosocial interventions to enhance internal validity and sample homogeneity, as recommended for youth sport research focusing on psychological and physiological outcomes (Malina et al., 2015; R. S. Lloyd et al., 2014).

### 2.2. Procedure and Data Analysis

Prior to the commencement of the study, all anthropometric measurements were carefully planned and standardized to ensure accuracy and reliability. Measurements were conducted at the ISSEP Ksar Saïd laboratory under controlled environmental conditions, with temperature maintained between 20 and 24 °C and relative humidity between 40 and 60%. Each assessment session was scheduled between 09:00 and 12:00 (midday) to minimize the influence of diurnal variation on body composition and physical performance. Participants were instructed to wear light clothing and to remove shoes and accessories to allow precise measurement.

Anthropometric variables were assessed according to the International Society for the Advancement of Kinanthropometry (ISAK) protocols using a calibrated stadiometer for height, a digital scale for body mass, a non-stretchable tape measure for circumferences, and skinfold calipers for estimating body fat percentage (Esparza-Ros et al., 2019). All instruments were calibrated before each measurement session, and assessments were performed by trained personnel following standardized procedures to reduce inter- and intra-observer variability. These measures ensured that baseline anthropometric data were accurate, reliable, and comparable across participants, consistent with best practices in youth sport research (Marfell-Jones et al., 2012; Norton & Olds, 2001) and to ensure that baseline anthropometric data were accurate and comparable across participants.

**Figure 2:**
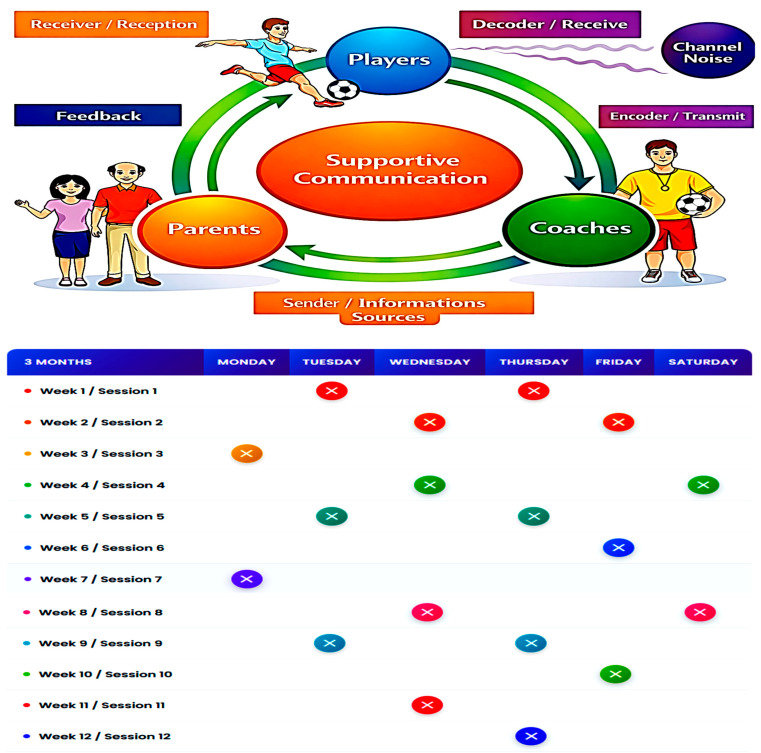
Conceptual Framework of Supportive Communication Between Parents, Coaches, and Players and Its 12-Session Intervention Schedule.

An official invitation was sent to the parents of all eligible players, on behalf of each club, to attend a meeting in which the study protocol was explained. This meeting was attended by football coaches and experts in social and sports psychology, during which parental consent for participation was obtained. The training program was structured over a 12-week period, consisting of 12 sessions (1 session per week), each held from 17:00 to 19:00. The ambient temperature during the sessions ranged from 20 °C to 25 °C. The intervention included a supportive communication component, with 20 min allocated before each session (Figure 2). The sessions took place in different locations depending on the group assignment, Group 1 (SCCP) received supportive communication from parents, coaches, and psychological specialists in the locker rooms; Group 2 (SCC) received supportive communication from coaches and psychological specialists in the locker rooms; Group 3 (SCP) received supportive communication from parents; and Group 4 (Control) received no structured communication intervention. All communication sessions followed a standardized protocol to ensure consistency in content and delivery across groups, thereby minimizing potential implementation bias and improving internal validity.

The supportive communication intervention was delivered in a fully standardized manner across all sessions and groups. At each session, coaches and psychological experts followed pre-defined scripts and structured practical exercises to convey consistent messages to both parents and coaches. The timing, content, and style of communication were strictly guided to ensure uniform exposure and reduce variability in delivery. All sessions were conducted according to the intervention manual, which was developed based on established frameworks for psychosocial interventions in youth sport (Kazdin, 2021; Michie et al., 2005). The manual included session objectives, step-by-step guidelines, example messages, interactive exercises, and monitoring checklists to ensure fidelity and reproducibility across different deliverers and to minimize the risk of bias due to inconsistent implementation and enhanced the internal validity of observed psychological and behavioral outcomes.

The actual training sessions were held on a football field with dimensions of 120 m by 60 m, incorporating a variety of physical, technical, and tactical exercises. prior to the start of the program. Anxiety, stress, and depression levels were assessed using the DASS-21 scale both before (session 1) and after (session 12). Additionally, self-regulation levels were measured using the Self-Regulation Questionnaire (SRQ) at the beginning and conclusion of the study.

Session 1—Trust: Build trust among players, coaches, and parents through personal stories and small-sided games (3v3, 4v4). Cool-down reflection links trust to performance.

Session 2—Goal Setting: Set short- and long-term goals. Rondo drills (5v2, 4v2) enhance decision-making and ball retention. Players reflect on goal achievement.

Session 3—Positive Reinforcement: Focus on effort and perseverance. Players give positive feedback during 4v4 games. Cool-down includes breathing exercises and reflection.

Session 4—Self-Regulation: Manage emotions under stress with deep breathing and role-play. Training emphasizes defensive transitions and high-pressure situations. Reflection on emotional control.

Session 5—Resilience: Learn mental toughness through stories of overcoming setbacks. Shooting drills under pressure simulate game scenarios. Reflection on persistence and handling mistakes.

Session 6—Teamwork: Emphasize collaboration, communication, and support. Passing drills (groups of 3) and 7v7 games focus on team structure. Yoga and reflection during cool-down.

Session 7—Stress Management: Teach relaxation, visualization, and focus techniques. Counter-attacking drills and fast breaks simulate pressure. Meditation and reflection reinforce stress control.

Session 8—Gratitude: Express appreciation for teammates, coaches, and parents. Positional play drills reinforce collaboration and recognition of effort. Reflection on gratitude’s impact.

Session 9—Overcoming Mistakes: Normalize mistakes as learning opportunities. Passing patterns and finishing drills emphasize composure and lessons learned.

Session 10—Leadership: Develop responsibility and leadership on and off the field. 9v9 tactical games allow players to direct the team and make key decisions. Reflection on leadership moments.

Session 11—Confidence: Recognize individual strengths and leverage them in competitive drills (crossing and finishing under pressure). Reflection on how confidence affects performance.

Session 12—Reflection & Progress Review: Review overall progress, celebrate achievements, and apply learned strategies in a full-field game. Group reflection sets goals for continued growth (Table 1).

### 2.3. Measures

#### 2.3.1. Depression Anxiety Stress Scale-21 (DASS-21)

The Arabic version of (DASS-21) validated by (Ali et al., 2017), was used to evaluate depression, anxiety, and stress among their study participants with reliability (Cronbach’s alpha = 0.87). It is a well-established self-report instrument that measures the severity of these three related emotional states. The DASS-21 consists of three subscales, each with 7 items, which assess depression, anxiety, and stress. Respondents are asked to rate the extent to which they have experienced each symptom over the past week on a 4-point scale ranging from 0 (Never = did not apply to me at all), 1 (SOMETIMES = Applied to me to some degree, or some of the time) 2, (OFTEN = Applied to me to a considerable degree, or a good part of time) to 3 (ALMOST ALWAYS = applied to me very much or most of the time). Higher scores on each subscale indicate greater levels of depression, anxiety, or stress. Previous confirmatory factor analyses of the Arabic version supported the original three-factor structure (CFA fit indices: CFI = 0.95, TLI = 0.94, RMSEA = 0.06), confirming factorial validity in adolescent and young adult populations.

#### 2.3.2. Self-Regulation Questionnaire (SRQ)

The Arabic version of the Self-Regulation Questionnaire (SRQ), adapted by Aitcheson et al. (2017), was used to investigate a level of self-regulation for participants. The Arabic version retains the key characteristics of the original version while making necessary linguistic and cultural adjustments for Arabic-speaking populations, it measures self-regulation, including an individual’s ability to set goals, monitor progress, and regulate behavior and emotions in achieving long-term objectives. The SRQ consists of 63 items rated on a Likert-type scale (typically ranging from 1 = “Strongly disagree” to 5 = “Strongly agree”) and covers multiple dimensions of self-regulation such as Receiving, Planning, Evaluating, Triggering, Searching and Implementing, and with items grouped into various subscales, providing insights into how well respondents manage tasks, maintain self-control, and stay focused on their goals; the adaptation process ensured the questionnaire is both culturally and linguistically appropriate for Arab populations, preserving the meaning while respecting local nuances, and the Arabic SRQ has demonstrated strong internal consistency, reliability, and construct validity, making it a valuable tool for researchers and practitioners working with Arabic-speaking populations to understand how individuals regulate their thoughts, emotions, and behaviors in diverse settings. The Arabic SRQ demonstrated satisfactory reliability (Cronbach’s α = 0.74–0.83) and its factorial validity has been confirmed in previous studies (CFA fit indices: CFI = 0.93, TLI = 0.92, RMSEA = 0.07), supporting its use for evaluating self-regulation in Arabic-speaking adolescents and young adults.

### 2.4. Data Analysis

Data were expressed as means and standard deviations (M ± SD). The Shapiro–Wilk test was used to assess the normality of the distribution for all variables. A 4 (groups) × 2 (time points: before and after) repeated measures analysis of variance (ANOVA) was performed to analyze levels of depression, anxiety, stress, and self-regulation. The assumption of sphericity was checked using the Mauchly test, and if it was violated, the Greenhouse-Geisser correction was applied. Effect sizes were expressed as partial eta squared (η^2^p), with values of 0.01, 0.06 and 0.14 representing small, medium and large effects, respectively. For significant main effects or interactions, a Tukey’s Honestly Significant Difference (HSD) post hoc test was performed to identify specific differences between groups or time points. In addition, linear mixed-effects models (LMMs) with 95% confidence intervals (95% CI [lower-upper]) were used to account for repeated measures and random effects, including variance components for club-level and residual-level variability, providing robust estimates of group, time, and interaction effects while handling inter-participant and inter-club differences. The (%) percentage change for each variable between pre- and post-intervention was calculated using the formula: [(Post score − Pre-score)/Pre-score] × 100. The level of statistical significance was set at *p* < 0.05. All statistical analyses were performed using IBM SPSS Statistics version 29.0 (IBM Corp., Armonk, NY, USA).

## 3. Results

### 3.1. Depression, Anxiety and Stress Scale-21

Results presented in Table 2 illustrate the effects of the intervention on mood state variables, as assessed using repeated-measures analysis of variance (ANOVA) and percentage changes between pre- and post-intervention measurements.

#### 3.1.1. Depression

Findings showed a highly significant effect of the intervention on depression scores. There was a strong time effect (F = 780.94, *p* < 0.001, ηp^2^ = 0.93 [Very large]), indicating a substantial reduction in depression from pre- to post-intervention across all participants. The group effect was also significant (F = 45.79, *p* < 0.001, ηp^2^ = 0.71 [Very large]), reflecting meaningful differences between the four groups regardless of time. Moreover, the time × group interaction was significant (F = 117.97, *p* < 0.001, ηp^2^ = 0.86 [Very large]), suggesting that the magnitude of depression reduction differed considerably among groups. The percentage (%) changes indicate that interventions involving both coach and parent support produce the greatest improvement in depressive symptoms, whereas single-source support or no intervention results in smaller changes. The SCCP group demonstrated a reduction (−45.77%), followed by SCC group (−23.76%) and SCP group (−13.54%), while the CG showed the smallest reduction (−4.97%).

#### 3.1.2. Anxiety

For anxiety, the analysis revealed a significant time effect (F = 251.81, *p* < 0.001, ηp^2^ = 0.81 [Very large]), showing a large decrease in anxiety scores from pre- to post-intervention. There was a significant group effect (F = 25.32, *p* < 0.001, ηp^2^ = 0.57 [Very large]), indicating that anxiety levels differed between the four groups across both time points. The time × group interaction was also statistically significant (F = 35.50, *p* < 0.001, ηp^2^ = 0.65 [Very large]), highlighting that the reduction in anxiety was not uniform and depended on the specific intervention condition. The percentage changes show that the combined supportive communication from both coach and parent SCCP group (−46.55%) was the most effective in reducing anxiety, followed by SCC group (−32.96%) and SCP group (−25.01%). In contrast, the CG showed a slight increase in anxiety (+0.58%).

#### 3.1.3. Stress

Stress scores demonstrated the most pronounced changes. The time effect was significant (F = 2888.67, *p* < 0.001, ηp^2^ = 0.98 [Very large]), indicating a decrease in stress levels from pre- to post-intervention. The group effect was significant (F = 22.68, *p* < 0.001, ηp^2^ = 0.54 [Very large]), showing notable differences between groups regardless of time. Finally, the time × group interaction was significant (F = 268.09, *p* < 0.001, ηp^2^ = 0.93 [Very large]), demonstrating that stress reduction varied across the four groups, with certain interventions having a more pronounced effect. The % changes indicate that the SCCP group (−48.29%) was the most effective in reducing stress, The SCC group (−37.59%) and SCP group (−23.80%). The CG showed a modest reduction (−7.75%).

Tukey post hoc comparisons revealed that SCCP produced significantly more favorable mood outcomes than both SCP and CG across all three outcome variables; complete pairwise comparison statistics for all six group dyads per outcome are available from the corresponding author upon request. For depression, SCCP demonstrated significantly lower scores than SCP (*p* = 0.001, 95% CI: [−7.200 to −3.205]) and CG (*p* = 0.001, 95% CI: [−6.602 to −3.405]), while SCC also exhibited significantly lower depression than SCP (*p* < 0.001, 95% CI: [−5.375 to −2.520]) and CG (*p* < 0.001, 95% CI: [−5.197 to −2.299]). A similar pattern emerged for anxiety, with SCCP demonstrating significantly lower scores than SCP (*p* < 0.001, 95% CI: [−5.827 to −2.204]) and CG (*p* = 0.002, 95% CI: [−5.482 to −1.165]), and SCC exhibiting significantly lower anxiety than SCP (*p* < 0.001, 95% CI: [−5.032 to −1.524]) and CG (*p* = 0.001, 95% CI: [−4.044 to −1.128]). For stress, SCCP again demonstrated significantly lower scores than SCP (*p* < 0.001, 95% CI: [−5.088 to −1.568]) and CG (*p* < 0.001, 95% CI: [−3.692 to −1.071]), while SCC was significantly lower than SCP (*p* < 0.001, 95% CI: [−4.742 to −1.471]) and CG (*p* < 0.001, 95% CI: [−3.077 to −1.243]). Across all three outcomes, the hierarchical gradient of intervention efficacy, with SCCP producing the largest reductions, followed by SCC, SCP, and CG respectively, was consistently maintained, corroborating the pattern observed in the percentage change data reported in Table 2.

**Figure 3:**
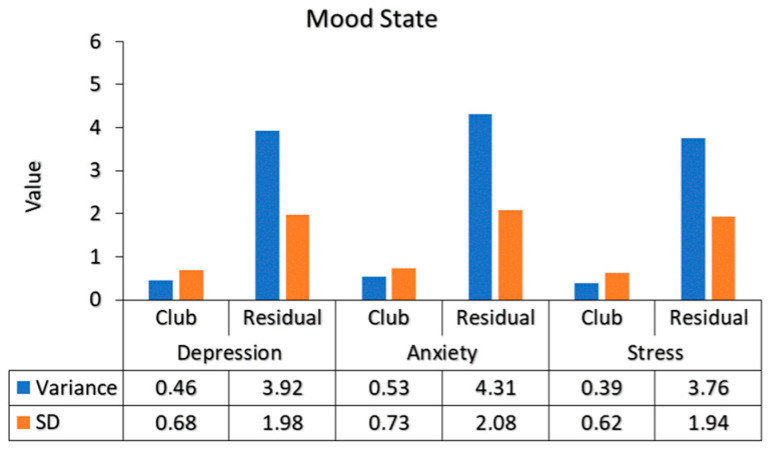
Variance and Standard Deviation of Mood State Variables Between Club and Residual Components.

To account for the potential non-independence of observations arising from recruiting participants from four different football clubs, we conducted supplementary analyses using linear mixed-effects models with club included as a random intercept. Figure 3 presents the variance components for each mood state variable. The variance attributable to club membership was small relative to the residual variance for all outcomes (Depression: 0.46 vs. 3.92; Anxiety: 0.53 vs. 4.31; Stress: 0.39 vs. 3.76), indicating that differences between clubs contributed minimally to the overall variability. Most of the variation in depression, anxiety, and stress scores occurred at the individual level rather than the club level.

Result in Table 3 presents the mixed-effects regression results examining the effects of time, group, and their interactions on depression, anxiety, and stress. For depression, a significant reduction over time was observed (β = −1.02, *p* = 0.001, 95% CI [−1.63, −0.41]), with the greatest improvement in the SCCP group receiving combined parental and coach support (Time × SCCP: β = −7.65, *p* < 0.001, 95% CI [−8.79, −6.51]). SCC and SCP groups also showed significant time interactions, though smaller (Time × SCC: β = −3.26; Time × SCP: β = −1.88). Anxiety did not significantly change overall (Time: β = 0.12, *p* = 0.757), but group-time interactions revealed strong reductions in SCCP (β = −10.63, *p* < 0.001), SCC (β = −7.25, *p* < 0.001), and SCP groups (β = −6.22, *p* < 0.001), highlighting that combined or single-source support effectively reduced anxiety. For stress, both main effect of time (β = −1.60, *p* < 0.001) and interaction effects were significant, with the largest decrease in SCCP group (Time × SCCP: β = −9.52, *p* < 0.001), followed by SCC group (β = −6.59) and SCP group (β = −4.02). Across outcomes, SCCP consistently produced the largest improvements, indicating that dual supportive communication from parents and coaches substantially enhances mental health indicators compared to support from a single source or control.

### 3.2. Self-Regulation Questionnaire

Results in Table 4 showed significant effects for time, group, and their interaction in key self-regulation variables. For Planning, there were significant time (F = 373.00, *p* < 0.001, ηp^2^ = 0.86 [Very large]), group (F = 39.65, *p* < 0.001, ηp^2^ = 0.68 [Very large]), and interaction (F = 16.97, *p* < 0.001, ηp^2^ = 0.47 [Very large]) effects. Similar patterns were found in Implementing time (F = 235.34, *p* < 0.001, ηp^2^ = 0.80 [Very large]), group (F = 6.31, *p* < 0.001, ηp^2^ = 0.25 [Very large]), interaction (F = 23.49, *p* < 0.001, ηp^2^ = 0.55 [Very large]) and Triggering time (F = 38.73, *p* < 0.001, ηp^2^ = 0.40 [Very large]), group (F = 22.84, *p* < 0.001, ηp^2^ = 0.55 [Very large]), and interaction (F = 28.44, *p* < 0.001, ηp^2^ = 0.60 [Very large]). Moderate effects were observed for Receiving and Evaluating, whereas Formulating and Searching showed smaller and non-significant effects. The percentage change between pre- and post-intervention shows that the SCCP group achieved the highest improvements across all self-regulation variables, with gains ranging from +14.48% in Searching to +66.38% in Planning. SCC showed moderate increases, notably +34.29% in Planning and +13.79% in Receiving. SCP had smaller improvements, ranging from −1.74% in Searching to +38.31% in Planning, with minimal change in Triggering (+0.39%). The CG demonstrated the lowest changes, ranging from 0.00% in Triggering to +36.65% in Planning.

The post hoc Tukey comparisons between groups for self-regulation variables revealed several significant differences. For Planning, SCCP significantly outperformed SCC, SCP, and CG (*p* < 0.001, 95% CI; [0.439–0.904]), while SCC scored lower than SCCP (*p* < 0.001, 95% CI; [−0.727 to −0.439]) and CG (*p* = 0.011, 95% CI; [0.054–0.353]). In Implementing, SCCP exceeded SCC (*p* < 0.001, 95% CI; [0.555–1.551]) and CG (*p* = 0.002, 95% CI; [0.403–1.474]), and SCC scored lower than SCCP (*p* < 0.001, 95% CI; [−1.551 to −0.555]). For Formulating, SCCP was higher than SCC (*p* = 0.028, 95% CI; [0.069–1.015]) and CG (*p* = 0.004, 95% CI; [0.272–1.176]), while CG was lower than SCCP (*p* = 0.004, 95% CI; [−1.176 to −0.272]). Receiving showed SCCP superiority over SCC (*p* = 0.010, 95% CI; [0.205–1.256]) and CG (*p* = 0.003, 95% CI; [0.381–1.477]), with CG lower than SCCP (*p* = 0.003, 95% CI; [−1.477 to −0.381]). In Evaluating, SCCP was significantly higher than CG (*p* = 0.002, 95% CI; [0.420–1.529]) and CG also differed from SCC (*p* = 0.024, 95% CI; [−1.078 to −0.089]). For Searching, SCCP differed from CG (*p* = 0.017, 95% CI; [0.114–0.973]) and CG scored substantially higher than SCCP and SCC in some comparisons (*p* < 0.001, 95% CI; [1.071–3.692 and 1.243–3.077]). Finally, in Triggering, SCCP exceeded SCC, SCP, and CG (*p* < 0.001, 95% CI; [0.619–2.097]), with CG lower than SCCP and SCC (*p* < 0.03, 95% CI; [−2.097 to −0.106]).

Findings in Table 5 show the effects of time, group, and their interactions on seven self-regulation sub-domains: Planning, Implementing, Formulating, Receiving, Evaluating, Searching, and Triggering. Planning significantly improved over time (β = 1.52, *p* < 0.001, 95% CI [1.06, 1.98]), with the SCCP group showing the largest time interaction effect (Time × SCCP: β = 1.99, *p* < 0.001, 95% CI [0.85, 3.13]), indicating enhanced planning ability when both parental and coach support were provided. Implementing showed a significant improvement in the SCCP group interaction (Time × SCCP: β = 1.60, *p* < 0.001, 95% CI [0.58, 2.62]), despite the main time effect being marginal (*p* = 0.064). Triggering also increased over time and in SCCP (Time × SCCP: β = 1.41, *p* < 0.001, 95% CI [0.57, 2.25]). Other sub-domains, Formulating, Receiving, Evaluating, and Searching, did not show significant main or interaction effects except for Evaluating in SCCP (Time × SCCP: β = 1.09, *p* = 0.044, 95% CI [0.05, 2.13]).

**Figure 4:**
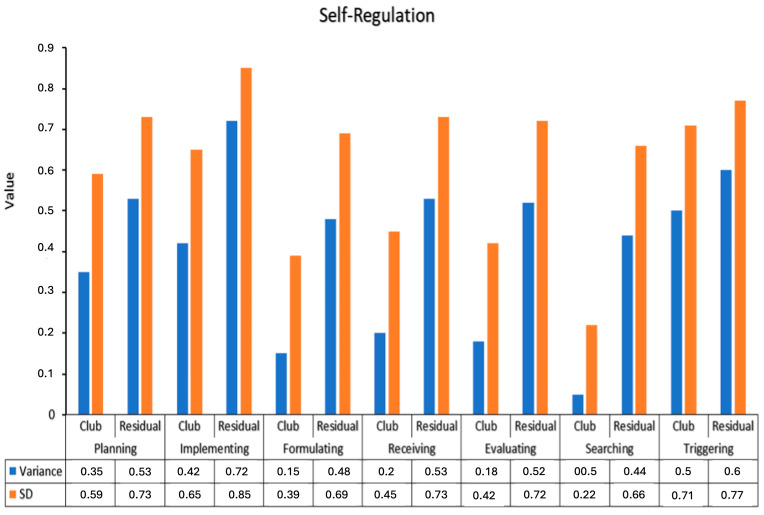
Variance and Standard Deviation of Self-Regulation Sub-Domains Across Club and Residual Measures.

Results in Figure 4 displays variance and standard deviation (SD) for seven self-regulation sub-domains: Planning, Implementing, Formulating, Receiving, Evaluating, Searching, and Triggering, comparing club-based and residual measures. Across all sub-domains, residual measures consistently show higher variance and SD than club measures, indicating greater variability in self-regulation outcomes beyond the structured club environment. The largest differences were observed in Implementing (Variance: 0.42–0.72; SD: 0.65–0.85), Triggering (Variance: 0.50–0.60; SD: 0.71–0.77), and Planning (Variance: 0.35–0.53; SD: 0.59–0.73). In contrast, Searching and Formulating showed relatively smaller variance differences between club and residual scores.

## 4. Discussion

This study examined the effects of a multi-component psychosocial program that included structured supportive communication strategies delivered by both parents and coaches on adolescent athletes’ mental health and self-regulation. Consistent with our hypotheses, the group receiving combined parent- and coach-based support (SCCP) showed larger improvements in depression, anxiety, stress, and self-regulation than the single-source or control groups. These findings highlight the added value of multi-source, integrated support in fostering psychological well-being among youth athletes. Given the magnitude of some observed effects, particularly for self-reported outcomes, these findings should be interpreted cautiously. Expectancy effects, shared-method variance, and the intervention’s focus on psychological skills may have contributed to the observed changes.

Supportive communication was a central component of the intervention framework and is theoretically linked to emotional security during adolescence (Healy et al., 2024; Sahi et al., 2023). However, because communication quality and dosage were not measured directly, the present findings should be interpreted as reflecting the effects of the overall program rather than communication processes alone. Open, empathetic, and responsive interactions with parents and coaches help adolescents feel understood and connected, reducing feelings of isolation and mitigating internalizing symptoms (De Raeymaecker & Dhar, 2022; Kim & Park, 2020; A. Lloyd et al., 2023; Ratliff et al., 2023). The observed improvements in mental health outcomes in the SCCP group are consistent with theoretical models emphasizing the buffering role of social support in stress management (Acoba, 2024; Davis et al., 2019). When adolescents perceive that significant adults in their environment provide affirmation and guidance, they are more likely to reappraise stressors as manageable challenges rather than threats, resulting in decreased psychological and physiological stress responses (Cohen & Wills, 1985).

Emotion regulation is a plausible explanatory pathway linking psychosocial support to mental health outcomes (Morris et al., 2007). However, because self-regulation was not formally tested as a mediator, these interpretations should be considered theoretical rather than causal. Adolescents who are able to plan, implement, monitor, and adjust their behaviors effectively demonstrate greater resilience in the face of stress (Cui et al., 2014). Cognitive reappraisal, a key strategy in emotion regulation, allows athletes to reinterpret potentially negative events in a more adaptive light (Gomes et al., 2022; Troy et al., 2018). For instance, coaches who frame mistakes as learning opportunities enable athletes to respond constructively rather than emotionally, reinforcing adaptive coping strategies. Observing consistent supportive behaviors from both parents and coaches provides models for healthy emotional regulation, enhancing adolescents’ capacity to self-regulate across different contexts (Maurice et al., 2021).

Self-efficacy may represent another important mechanism through which multi-component psychosocial interventions influence mental health, but this pathway was not empirically tested in the present study. Interventions emphasizing effort, persistence, and skill development rather than outcomes fostered adolescents’ belief in their ability to manage challenges effectively (Mata et al., 2021). This heightened sense of competence not only reduced feelings of helplessness and inadequacy but also encouraged proactive engagement in problem-solving, further supporting emotional and behavioral regulation (Warner & Budd, 2018). The combination of improved self-regulation and enhanced self-efficacy likely contributed to the statistically large reductions in depression, anxiety, and stress observed in the SCCP group (Chan et al., 2012; Huang et al., 2023).

The pattern of findings across SRQ subscales further indicates that self-regulation did not improve uniformly. While dimensions related to monitoring, adjusting, and evaluating showed clearer improvements, other components such as searching and formulating were less responsive to the intervention. This divergence is theoretically consistent with models of self-regulated behavior, which distinguish between emotion- and feedback-dependent regulation processes and more cognitively driven planning processes (Mcgreary et al., 2024).The latter may require longer exposure, task-specific practice, or explicit goal-structuring to change, and may therefore be less sensitive to short-term psychosocial interventions. These results suggest that the intervention selectively influenced certain facets of self-regulation rather than producing global improvement across all dimensions.

The implications of these findings extend beyond individual mental health outcomes. Adolescents experiencing reduced psychological distress are more likely to remain engaged in sports, showing higher motivation, commitment, and resilience. By integrating supportive communication strategies into coaching and training programs, educators and sports organizations can foster environments that simultaneously prioritize athletic development and psychological well-being. Furthermore, training modules for coaches and parents could emphasize effective communication techniques, emotional support strategies, and awareness of mental health issues, establishing systemic frameworks that ensure holistic athlete care.

From a broader perspective, these findings suggest potential applied relevance for how psychosocial support is structured in youth sport settings. While the present study was not designed to inform policy directly, youth sport organizations may consider integrating communication-focused and mental health–oriented training for coaches and parents as part of broader athlete development frameworks. Such approaches could help foster environments that are more attentive to young athletes’ psychological well-being, which is increasingly recognized as important for sustained engagement, motivation, and long-term development. However, the translation of these findings into formal organizational or policy guidelines should await confirmation from larger, multi-site studies using objective and multi-method outcome assessments.

Future research should explore the longitudinal effects of combined parental and coach interventions, including their durability over extended periods and applicability across various sports and cultural contexts. Investigating potential moderators, such as individual differences in temperament, baseline psychological functioning, or prior athletic experience, could provide further insight into how these interventions can be tailored for maximum effectiveness. Additionally, examining the interplay between sportive communication, self-efficacy, and specific aspects of self-regulation may clarify the mechanisms underlying observed mental health improvements. Future studies should explicitly measure communication quality and test mediation models to determine whether self-regulation and self-efficacy explain observed improvements.

### Limitations

The study has several limitations that should be considered when interpreting the findings. First, the research focused on a specific cohort of male adolescent soccer players, which may limit the generalizability of the results to other sports, age groups, or to non-athlete populations. The exclusive inclusion of male athletes also precludes examination of potential gender-specific responses to psychosocial interventions, as female athletes may differ in emotional processing, communication sensitivity, and stress regulation.

In addition, the study’s short-term design restricts conclusions about the long-term sustainability of the observed improvements in mental health, and future research should examine whether these effects persist over extended periods. Cultural and contextual factors influencing communication styles were not fully explored, which may further affect the transferability of the findings across different settings.

Moreover, although the intervention was designed around supportive communication, communication quality and dosage were not measured directly, and key hypothesized mechanisms—including self-regulation and self-efficacy—were not tested as mediators. This limits causal inference regarding the processes through which the intervention exerted its effects. Finally, the study focused primarily on verbal supportive communication and did not account for other forms of parental and coach support, such as non-verbal behaviors, which may also contribute to adolescent athletes’ mental health.

## 5. Conclusions

This study provides preliminary evidence that a multi-component program involving parents and coaches is associated with improvements in adolescent athletes’ mental health and self-regulation. The findings suggest that coordinated support from significant adults in both the family and sport environment may contribute to reduced stress, anxiety, and depression, as well as to better psychological adjustment during adolescence.

By fostering emotional security, encouraging constructive communication, and supporting self-efficacy, such interventions may offer a promising framework for promoting psychological well-being in youth sport settings. However, these findings should be interpreted within the cultural and sporting context of the present sample and may not generalize to other populations or settings without further replication.

Importantly, the intervention was delivered with psychologist supervision to ensure fidelity and participant safety. Whether similar effects can be achieved in applied sport environments without professional oversight remains an important question for future research. Future studies should also examine the scalability, long-term sustainability, and cross-cultural applicability of such multi-component psychosocial interventions.

## Figures and Tables

**Table 1:** 12-Sessions Combined Supportive Communication (Coaches-Parents) and football training program.

Sessions	Supportive Communication Focus	Football Training Focus
**1**	Building Trust (positive language, team bonding)	Passing and receiving under pressure
**2**	Goal Setting (short and long-term focus)	Rondo exercises for decision-making and ball retention
**3**	Positive Reinforcement (acknowledging effort)	Small-sided games (4v4) with limited touches
**4**	Self-Regulation (handling stress, emotions)	Defensive transitions and pressing
**5**	Resilience (overcoming adversity)	Shooting accuracy Under pressure
**6**	Teamwork (importance of collaboration)	7v7 game focusing on communication and positioning
**7**	Managing Stress & Anxiety (relaxation techniques)	Counter-attacking and fast breaks
**8**	Gratitude (appreciating each other’s efforts)	Positional play under pressure
**9**	Overcoming Mistakes (learning from errors)	Defensive and attacking patterns
**10**	Leadership (responsibility, role modeling)	9v9 tactical game focusing on leadership
**11**	Confidence Building (emphasizing strengths)	Crossing and finishing under pressure
**12**	Reflection & Progress Review (celebrating achievements)	Full-field game applying learned strategies

**Table 2:** Pre/Post (%) Changes and Repeated-Measures ANOVA Results for mood state Components Across Supportive Communication Groups and the Control Group.

Variable	Group (n = 60)		M ± SD	% Change	Time Effect			Group Effect			Interaction Effect (Time × Group)		
					F	*p*≤	ηp^2^	F	*p*≤	ηp^2^	F	*p*≤	ηp^2^
**Depression**	**SCCP**	**Pre**	18.95 ± 1.97	−45.77%	780.94	<0.001	0.93	45.79	<0.001	0.71	117.97	<0.001	0.86
		**Post**	10.28 ± 2.15										
	**SCC**	**Pre**	18.01 ± 1.30	−23.76%									
		**Post**	13.73 ± 1.39										
	**SCP**	**Pre**	21.26 ± 1.40	−13.54%									
		**Post**	18.38 ± 2.26										
	**CG**	**Pre**	20.12 ± 0.99	−4.97%									
		**Post**	19.12 ± 0.79										
**Anxiety**	**SCCP**	**Pre**	22.58 ± 2.06	−46.55%	251.81	<0.001	0.81	25.32	<0.001	0.57	35.50	<0.001	0.65
		**Post**	12.07 ± 2.44										
	**SCC**	**Pre**	21.63 ± 1.05	−32.96%									
		**Post**	14.50 ± 2.10										
	**SCP**	**Pre**	24.39 ± 1.70	−25.01%									
		**Post**	18.29 ± 2.95										
	**CG**	**Pre**	20.59 ± 0.61	+0.58%									
		**Post**	20.71 ± 2.60										
**Stress**	**SCCP**	**Pre**	23.03 ± 1.74	−48.29%	2888.67	<0.001	0.98	22.68	<0.001	0.54	268.09	<0.001	0.93
		**Post**	11.91 ± 2.01										
	**SCC**	**Pre**	21.79 ± 0.99	−37.59%									
		**Post**	13.60 ± 1.23										
	**SCP**	**Pre**	23.61 ± 1.24	−23.80%									
		**Post**	17.99 ± 2.05										
	**CG**	**Pre**	20.65 ± 0.59	−7.75%									
		**Post**	19.05 ± 0.44										

**Note.** M ± SD = means and standard deviation; SCCP = supportive communication from parents and coaches; SCC = supportive communication from coaches only; SCP = supportive communication from parents only; CG = control group; % change = percentage change between pre and post intervention; F = F-Value; *p* = *p* value; ηp^2^ = partial eta square (effect size).

**Table 3:** Mixed-Effects Model Estimates for Depression, Anxiety, and Stress Across Groups and Time.

Outcome	Effect	Estimate (β)	SE	T	*p*-Value	95% CI
**Depression**	Intercept	20.11	0.42	47.88	<0.001	[19.29, 20.93]
	Time (Post)	−1.02	0.31	−3.29	0.001	[−1.63, −0.41]
	SCCP	−0.89	0.45	−1.98	0.049	[−1.77, −0.01]
	SCC	−0.44	0.43	−1.02	0.310	[−1.29, 0.41]
	SCP	1.14	0.47	2.43	0.017	[0.22, 2.06]
	Time × SCCP	−7.65	0.58	−13.19	<0.001	[−8.79, −6.51]
	Time × SCC	−3.26	0.55	−5.93	< 0.001	[−4.34, −2.18]
	Time × SCP	−1.88	0.60	−3.13	0.002	[−3.06, −0.70]
**Anxiety**	Intercept	20.60	0.46	44.78	<0.001	[19.69, 21.51]
	Time (Post)	0.12	0.39	0.31	0.757	[−0.65, 0.89]
	SCCP	1.99	0.52	3.83	<0.001	[0.97, 3.01]
	SCC	1.04	0.50	2.08	0.039	[0.06, 2.02]
	SCP	3.80	0.55	6.91	<0.001	[2.72, 4.88]
	Time × SCCP	−10.63	0.69	−15.41	<0.001	[−11.99, −9.27]
	Time × SCC	−7.25	0.66	−10.99	<0.001	[−8.55, −5.95]
	Time × SCP	−6.22	0.71	−8.76	<0.001	[−7.62, −4.82]
**Stress**	Intercept	20.71	0.39	53.10	<0.001	[19.94, 21.48]
	Time (Post)	−1.60	0.28	−5.71	<0.001	[−2.15, −1.05]
	SCCP	2.38	0.44	5.41	<0.001	[1.52, 3.24]
	SCC	1.14	0.42	2.71	0.008	[0.32, 1.96]
	SCP	2.96	0.46	6.43	<0.001	[2.06, 3.86]
	Time × SCCP	−9.52	0.51	−18.67	<0.001	[−10.52, −8.52]
	Time × SCC	−6.59	0.49	−13.45	<0.001	[−7.55, −5.63]
	Time × SCP	−4.02	0.53	−7.58	<0.001	[−5.06, −2.98]

**Note.** SCCP = supportive communication from parents and coaches; SCC = supportive communication from coaches only; SCP = supportive communication from parents only; β = unstandardized regression coefficient; SE = standard error; T = t-statistic; 95% CI = 95% confidence interval.

**Table 4:** Pre/Post (%) Changes and Repeated-Measures ANOVA Results for Self-Regulation Components Across Supportive Communication Groups.

Variable	Group (n = 60)		M ± SD	% Change	Time Effect			Group Effect			Interaction Effect (Time × Group)		
					F	*p*≤	ηp^2^	F	*p*≤	ηp^2^	F	*p*≤	ηp^2^
**Planning**	**SCCP**	**Pre**	2.29 ± 0.36	+66.38%	373.00	<0.001	0.86	39.65	<0.001	0.68	16.97	<0.001	0.47
		**Post**	3.81 ± 0.23										
	**SCC**	**Pre**	2.10 ± 0.51	+34.29%									
		**Post**	2.82 ± 0.17										
	**SCP**	**Pre**	2.01 ± 0.27	+38.31%									
		**Post**	2.78 ± 0.17										
	**CG**	**Pre**	1.91 ± 0.18	+36.65%									
		**Post**	2.61 ± 0.23										
**Implementing**	**SCCP**	**Pre**	3.17 ± 0.86	+35.33%	235.34	<0.001	0.80	6.31	<0.001	0.25	23.49	<0.001	0.55
		**Post**	4.29 ± 0.60										
	**SCC**	**Pre**	2.46 ± 0.58	+17.48%									
		**Post**	2.89 ± 0.47										
	**SCP**	**Pre**	2.85 ± 0.90	+22.11%									
		**Post**	3.48 ± 0.82										
	**CG**	**Pre**	2.68 ± 0.83	+8.58%									
		**Post**	2.91 ± 0.75										
**Formulating**	**SCCP**	**Pre**	3.42 ± 1.02	+17.84%	5.25	0.026	0.08	2.53	0.066	0.12	0.80	0.49	0.04
		**Post**	4.03 ± 0.82										
	**SCC**	**Pre**	3.10 ± 1.00	+5.16%									
		**Post**	3.26 ± 0.51										
	**SCP**	**Pre**	3.13 ± 1.05	+10.22%									
		**Post**	3.45 ± 0.89										
	**CG**	**Pre**	2.95 ± 0.97	+3.39%									
		**Post**	3.05 ± 0.77										
**Receiving**	**SCCP**	**Pre**	3.00 ± 0.91	+34.67%	31.22	<0.001	0.35	4.34	0.008	0.18	3.17	0.031	0.14
		**Post**	4.04 ± 0.94										
	**SCC**	**Pre**	2.61 ± 0.85	+13.79%									
		**Post**	2.97 ± 0.71										
	**SCP**	**Pre**	2.83 ± 0.91	+16.26%									
		**Post**	3.29 ± 0.98										
	**CG**	**Pre**	2.45 ± 0.65	+11.84%									
		**Post**	2.74 ± 0.59										
**Evaluating**	**SCCP**	**Pre**	2.81 ± 0.98	+38.79%	24.02	<0.001	0.30	6.50	<0.001	0.25	2.87	0.044	0.13
		**Post**	3.90 ± 0.81										
	**SCC**	**Pre**	2.74 ± 0.78	+16.42%									
		**Post**	3.19 ± 0.63										
	**SCP**	**Pre**	3.15 ± 1.07	+13.33%									
		**Post**	3.57 ± 0.82										
	**CG**	**Pre**	2.27 ± 0.69	+9.69%									
		**Post**	2.49 ± 0.66										
**Searching**	**SCCP**	**Pre**	2.90 ± 1.02	+14.48%	1.94	0.16	0.03	1.30	0.28	0.06	0.39	0.75	0.02
		**Post**	3.32 ± 1.12										
	**SCC**	**Pre**	2.73 ± 1.06	+6.96%									
		**Post**	2.92 ± 0.94										
	**SCP**	**Pre**	2.87 ± 1.04	−1.74%									
		**Post**	2.82 ± 0.23										
	**CG**	**Pre**	2.41 ± 0.84	+13.28%									
		**Post**	2.73 ± 0.83										
**Triggering**	**SCCP**	**Pre**	2.98 ± 0.92	+47.32%	38.73	<0.001	0.40	22.84	<0.001	0.55	28.44	<0.001	0.60
		**Post**	4.39 ± 0.42										
	**SCC**	**Pre**	2.39 ± 0.64	+7.11%									
		**Post**	2.56 ± 0.50										
	**SCP**	**Pre**	2.56 ± 0.83	+0.39%									
		**Post**	2.57 ± 0.57										
	**CG**	**Pre**	2.01 ± 0.50	0.00%									
		**Post**	2.01 ± 0.39										

**Note.** M ± SD = means and standard deviation; SCCP = supportive communication from parents and coaches; SCC = supportive communication from coaches only; SCP = supportive communication from parents only; CG = control group; % change = percentage change between pre and post intervention; F = F-Value; *p*≤ = *p* value; ηp^2^ = partial eta square (Effect Size).

**Table 5:** Mixed-Effects Model Estimates for Self-Regulation Sub-Domains Across Groups and Time.

Outcome	Effect	Estimate (β)	SE	T	*p*-Value	95% CI
**Planning**	Intercept	2.29	0.36	6.36	<0.001	[1.57, 3.01]
	Time (Post)	1.52	0.23	6.61	<0.001	[1.06, 1.98]
	SCCP	0.19	0.45	0.42	0.673	[−0.69, 1.07]
	SCC	−0.19	0.43	−0.44	0.658	[−1.05, 0.67]
	SCP	−0.28	0.47	−0.60	0.550	[−1.20, 0.64]
	Time × SCCP	1.99	0.58	3.43	<0.001	[0.85, 3.13]
	Time × SCC	0.72	0.55	1.31	0.192	[−0.34, 1.78]
	Time × SCP	0.77	0.60	1.28	0.203	[−0.42, 1.96]
**Implementing**	Intercept	3.17	0.86	3.68	<0.001	[1.44, 4.90]
	Time (Post)	1.12	0.60	1.87	0.064	[−0.05, 2.29]
	SCCP	0.71	0.58	1.22	0.225	[−0.43, 1.85]
	SCC	−0.43	0.47	−0.91	0.368	[−1.36, 0.50]
	SCP	0.63	0.82	0.77	0.443	[−0.97, 2.23]
	Time × SCCP	1.60	0.52	3.08	<0.001	[0.58, 2.62]
	Time × SCC	0.43	0.49	0.88	0.381	[−0.54, 1.40]
	Time × SCP	0.63	0.82	0.77	0.443	[−0.97, 2.23]
**Formulating**	Intercept	3.42	1.02	3.35	0.002	[1.36, 5.48]
	Time (Post)	0.61	0.82	0.74	0.465	[−1.03, 2.25]
	SCCP	0.32	0.52	0.62	0.538	[−0.72, 1.36]
	SCC	0.16	0.51	0.31	0.761	[−0.86, 1.18]
	SCP	0.32	0.89	0.36	0.723	[−1.42, 2.06]
	Time × SCCP	0.61	0.51	1.20	0.230	[−0.39, 1.61]
	Time × SCC	0.16	0.49	0.33	0.744	[−0.81, 1.13]
	Time × SCP	0.32	0.51	0.63	0.537	[−0.69, 1.33]
**Receiving**	Intercept	3.00	0.91	3.30	<0.001	[1.12, 4.88]
	Time (Post)	1.04	0.94	1.11	0.270	[−0.82, 2.90]
	SCCP	0.39	0.44	0.89	0.376	[−0.48, 1.26]
	SCC	−0.39	0.42	−0.93	0.354	[−1.21, 0.43]
	SCP	0.46	0.46	1.00	0.323	[−0.44, 1.36]
	Time × SCCP	1.04	0.58	1.79	0.074	[−0.10, 2.18]
	Time × SCC	0.36	0.49	0.73	0.466	[−0.60, 1.32]
	Time × SCP	0.46	0.50	0.92	0.362	[−0.54, 1.46]
**Evaluating**	Intercept	2.81	0.98	2.87	0.007	[0.91, 4.71]
	Time (Post)	1.09	0.81	1.35	0.183	[−0.51, 2.69]
	SCCP	0.07	0.44	0.16	0.872	[−0.80, 0.94]
	SCC	−0.07	0.42	−0.17	0.864	[−0.89, 0.75]
	SCP	0.32	0.82	0.39	0.698	[−1.29, 1.93]
	Time × SCCP	1.09	0.51	2.14	0.044	[0.05, 2.13]
	Time × SCC	0.45	0.49	0.92	0.362	[−0.53, 1.43]
	Time × SCP	0.42	0.51	0.82	0.416	[−0.60, 1.44]
**Searching**	Intercept	2.90	1.02	2.84	0.008	[0.92, 4.88]
	Time (Post)	0.42	1.12	0.37	0.716	[−1.78, 2.62]
	SCCP	0.19	0.51	0.37	0.715	[−0.83, 1.21]
	SCC	−0.17	0.49	−0.35	0.727	[−1.13, 0.79]
	SCP	−0.05	0.50	−0.10	0.921	[−1.05, 0.95]
	Time × SCCP	0.42	0.51	0.82	0.414	[−0.60, 1.44]
	Time × SCC	0.19	0.49	0.39	0.701	[−0.77, 1.15]
	Time × SCP	−0.05	0.50	−0.10	0.921	[−1.05, 0.95]
**Triggering**	Intercept	2.98	0.92	3.24	<0.001	[1.16, 4.80]
	Time (Post)	1.41	0.42	3.36	<0.001	[0.57, 2.25]
	SCCP	0.59	0.44	1.34	0.184	[−0.28, 1.46]
	SCC	−0.19	0.50	−0.38	0.708	[−1.18, 0.80]
	SCP	0.01	0.57	0.02	0.987	[−1.13, 1.15]
	Time × SCCP	1.41	0.42	3.36	<0.001	[0.57, 2.25]
	Time × SCC	0.17	0.50	0.34	0.736	[−0.81, 1.15]
	Time × SCP	0.01	0.57	0.02	0.987	[−1.13, 1.15]

**Note.** SCCP = supportive communication from parents and coaches; SCC = supportive communication from coaches only; SCP = supportive communication from parents only; β = unstandardized regression coefficient; SE = standard error; T = t-statistic; 95% CI = 95% confidence interval.

## Data Availability

Data are available from the first author upon reasonable request.
